# Provinols™, a Polyphenolic Extract of Red Wine, Inhibits In-Stent Neointimal Growth in Cholesterol-Fed Rabbit

**DOI:** 10.3390/pharmaceutics16101311

**Published:** 2024-10-09

**Authors:** Meyer Elbaz, Gérald Roul, Ramaroson Andriantsitohaina

**Affiliations:** 1 Department of Cardiology, Institute CARDIOMET, University Hospital of Toulouse, 31059 Toulouse, France; elbaz.m@chu-toulouse.fr; 2 Unité Fonctionnelle Dédiée à L’insuffisance Cardiaque, Pôle Médical et Chirurgical des Maladies Cardio-Vasculaires, Hôpitaux Universitaires de Strasbourg, 67000 Strasbourg, France; gerald.roul@unistra.fr; 3 PhyMedExp, University of Montpellier, Inserm, CNRS, 371 Avenue du Doyen G. Giraud, CEDEX 5, 34295 Montpellier, France

**Keywords:** polyphenols supplementation, stent, restenosis

## Abstract

**Background/Objectives:** Epidemiological studies indicate a potential correlation between the consumption of polyphenols and a reduced risk of developing cardiovascular disorders. The present study investigates the potential of a red wine polyphenol oral extract, Provinols™, to reduce neointimal hyperplasia following angioplasty in a hypercholesterolemic rabbit model. **Methods:** New Zealand white rabbits were fed 1% cholesterol-enriched chow for a period of eight weeks prior to the induction of iliac balloon injury and subsequent stent placement. Following the implantation of the stent, Provinols™ (20 mg/kg/day) or an identical placebo was administered orally for a period of four weeks in a randomized manner. Twenty-eight days following the stenting procedure, the arteries were harvested after euthanasia and subjected to histology assignment analysis. **Results:** The administration of Provinols™ did not result in a statistically significant change in either blood pressure or plasma cholesterol levels. However, Provinols™ treatment led to a notable reduction in the growth of the intima within the stented area, as well as a reduction in the thickness and surface area of the intima. It is of note that treatment with Provinols™ was associated with a reduction in the accumulation of fat within the arteries and a diminished inflammatory response to injury. **Conclusions:** The findings demonstrate that oral administration of Provinols™ has the potential to reduce in-stent neointimal growth and lipid deposition, likely due to its anti-inflammatory properties in iliac arteries from hypercholesterolemic rabbits. Additionally, these findings provide an evidence-based rationale for the potential therapeutic benefits of plant-derived polyphenols in the prevention of restenosis associated with stent placement.

## 1. Introduction

For over two decades, the preferred treatment of patients suffering from coronary artery disease is dilation of the coronary vessel with a catheter-borne balloon followed by stenting. Balloon angioplasty alone caused high restenosis rate, defined as excessive lumen narrowing of the treatment site, in about 30–50% of patients and is related to both intima hyperplasia and vascular remodeling. With regard to the latter, stenting represents a breakthrough in this field [1,2,3,4]. Drug-eluting stents (DES) were shown to inhibit in-stent neointimal hyperplasia and consequently were the most successful tool to decrease restenosis [5,6]. Nevertheless, several problems still remain: (i) DES treatment is only focal requiring multiple implantations in patients with multivessel disease with consequently a high cost, (ii) the efficacy and safety of DES might greatly differ depending on the stent delivery system and pharmacological agents used [7,8,9], (iii) late thrombosis associated with DES requires the use of a prolonged powerful antiplatelet therapy with an increased bleeding risk, although improved DES provide a reduction in in-stent restenosis, and (iv) preclinical studies have shown that local treatment could delay neointimal growth likely related to the nonbiodegradable polymer after elution of the drug [10,11]. Few studies using systemic drugs in the prevention of neointimal overgrowth have been reported including therapy of antiplatelet therapy, i.e., combination of clopidogrel and rivaroxaban [12], an inhibitor of neointimal formation with the selective inhibitor of monocyte chemotactic properties, bindarit [13], or anti-inflammatory drugs such as prednisone [14]. Probucol has been shown to have some interesting properties both in experimental and clinical studies [15]. However, potentially serious adverse effects and low tolerability have slowed down its development as a drug. Orally given sirolimus has shown promising results in a pilot study [16]. Nevertheless, studies on substitutive or adjunctive oral therapies remain limited. The putative molecule needs to fulfill the following criteria: ability to prevent neointimal hyperplasia, low cost and good safety profile. Moreover, an additional anti-inflammatory and anti-atheromatous effect could be of interest. Several lines of evidence have shown that dietary factors including polyphenols from red wine may fulfill the above-mentioned criteria. Indeed, they possess multiple biological properties including antioxidant, free radical–scavenging and anti-inflammatory properties, inhibition of platelet aggregation, vascular smooth muscle cell proliferation and migration [17,18]. Another relevant effect of polyphenols may be their ability to interact with the generation of nitric oxide from the vascular endothelium that leads not only to vasodilatation but also to the expression of genes protective for the cardiovascular system [19,20]. Therefore, we hypothesized that polyphenols extracted from red wine, owing to their potential, might prevent or at least significantly reduce in-stent neointimal hyperplasia in rabbit iliac arteries.

## 2. Materials and Methods

The investigation conformed to the guidelines of the American Physiological Society for the care and use of laboratory animals and with the recommendation from a consensus group for pre-clinical studies for device evaluation [21].

### 2.1. Stent Procedure and Tissue Harvest

Sixteen male New Zealand white rabbits (3 to 3.5 kg; Harlan Laboratories, Puteaux, France) were fed with chow containing 1% cholesterol for 8 weeks before implantation and 0.2% cholesterol after stent placement for 4 weeks. Each rabbit consumed on average 150 g chow per day. Eight weeks after the beginning of the diet, the animals were anesthetized (intramuscular ketamine 30 mg/kg and xylazine 5 mg/kg). The right carotid artery was dissected, isolated and then cannulated with a 5-F arterial lead. The lead was pushed to the abdominal aorta, under fluoroscopic guidance. Angiograms were obtained with a Philips 17-cm image intensifier. Two minutes after molsidomin administration (0.1 mg), right iliac artery balloon injury (conventional balloon angioplasty: 2.5 mm in diameter, 15 mm long; two 60-s inflation at a pressure of 6 atmospheres) was performed in anesthetized rabbits followed by placement of a BX Velocity stent (Cordis Inc. Johnson and Johnson, Lyon, France). Modus operandi of stent placement was classic: two 30-s balloon inflations; pressure was 8 to 12 atmospheres and stent to artery ratio of 1.1:1. The angioplasty procedure was performed after intravenous administration of heparin (100 IU/kg) and acetyl salicylic acid (ASA 10 mg/kg). When the time duration of the procedure was shorter than 15 min, the same protocol was applied to the left iliac artery. After the procedure, all animals received ASA 20 mg/d and clopidogrel 10 mg/d orally until euthanasia. Rabbits were randomly assigned to Provinols™ or placebo (control group). Provinols™ was diluted in water and given daily by oral administration at a dose of 20 mg/kg.

Twenty-eight days after stenting, animals were anesthetized, and a pre-euthanasia angiogram of the iliac artery was completed, followed by euthanasia and perfusion-fixation (4% paraformaldehyde).

### 2.2. Biochemical Parameters and Blood Pressure Measurements

Blood was withdrawn from the auricular vein (in some cases from the auricular artery) of all rabbits at baseline, following anesthesia but prior to stenting. A second blood test was conducted under a new anesthetic using the same methodology prior to euthanasia.

The blood was collected in ethylenediaminetetraacetic acid (EDTA-K2) tubes (BD, East Rutherford, NJ, USA), which contain an anticoagulant. Plasma was separated by centrifugation at 2000× *g* for 10 min at 4 °C. Alkaline phosphatase, glutamate transferase, aspartate aminotransferase (ASAT) and alanine aminotransferase (ALAT) activities were measured by routine methods using commercial kits on a Hitachi 902 autoanalyzer (Tokyo, Japan). Furthermore, total cholesterol, HDL cholesterol and triglycerides (TG) were determined using direct measurement assays.

Following catheterization of the carotid artery, blood pressure was measured and monitored at baseline and prior to euthanasia. The measurements were conducted under anesthesia. The intra-arterial catheter was connected to a sensor (Bioseb RS-IBP4, Vitrolles, France) using a three-way infusion valve and a system of tubing filled with heparinized physiological serum.

### 2.3. Data Analysis

Implanted sites were dehydrated in alcohol solutions of increasing concentrations, clarified in xylene and embedded in polymethylmethacrylate. For each stent, 3 sections were taken from the proximal, middle and distal portions. Sections were obtained by a microcutting and grinding technique adapted from Donath [22]. The sections were stained with modified Paragon staining for qualitative and quantitative analysis. Histological slides were blindly examined under light microscopy (Nikon Eclipse E600, fitted with lenses, coupled with a digital camera DN 100 Nikon, Tokyo, Japan). A semi-quantitative histological evaluation was performed according to the ISO 10993-6 standard [23]. Histological micrographs were performed for each specimen. Each analyzed parameter was graded according to a semi-quantitative scale (0 to 4; absent to severe). These parameters allowed an accurate evaluation of any inflammation, foreign body reaction, immunological reaction, or neointimal formation.

A quantitative histomorphometric analysis with computerized planimetry was also performed: All the sections were studied using a Zeiss Axioscope microscope fitted with different objectives and equipped with a color image analyzing system SAMBA^®^ (SAMBA Technologies, Meylan, France). The mean neointimal thickness (µm), residual lumen surface, theoretical lumen surface (corresponding to the initial lumen delimited by the internal elastic lamina), in-stent neointima and media surfaces were measured (mm^2^) and the stenosis percentage was calculated.

Stenosis percentage = 100 × (Neointima + Stent surfaces)/Theoretical lumen surface

The mean percentage of restenosis reduction for the Provinols™ group compared to control was calculated according to the following formula:

Mean percentage of restenosis reduction = 100 × (Mean percentage of stenosis of Control group − Mean percentage of stenosis of Provinols group)/Mean percentage of stenosis of Control group.

### 2.4. Statistical Analysis

Data are expressed as means ± SEM. Unpaired Student *t* test was used for between-group comparisons. A value of *p* < 0.05 was considered significant.

## 3. Results

### 3.1. Biochemical and Physiological Measurements

As shown in Table 1, plasma cholesterol levels were similar among the two groups both at the time of stent implantation and at the time of sacrifice. There were no significant differences in blood pressure between the two groups throughout the experiments.

Because of the high cholesterol regimen (1%) given during the first 8 weeks, we observed a weight loss (mean 0.25 ± 0.15 kg) and two deaths before stent implantation. Two other animals died, one in the Provinols™ group just after the procedure and one control 10 days after implantation. After stent implantation, Provinols™-treated rabbits regained more weight than their placebo-treated counterparts (0.43 kg versus 0.24 kg; NS). The angiographic control obtained before euthanasia revealed that all arteries were widely patent in the two groups.

### 3.2. Histological Qualitative Analysis

Eight stents deployed in six Provinols ™-treated animals were compared with eight stents in six placebo-treated ones (21 versus 20 sections). The stents were well deployed in both groups. Neither material-related adverse events around the stent struts nor obvious microscopic stent material degradation was observed in any of the specimens. The inner surface of all the samples was fully lined by endothelial-like cells.

**Figure 1:**
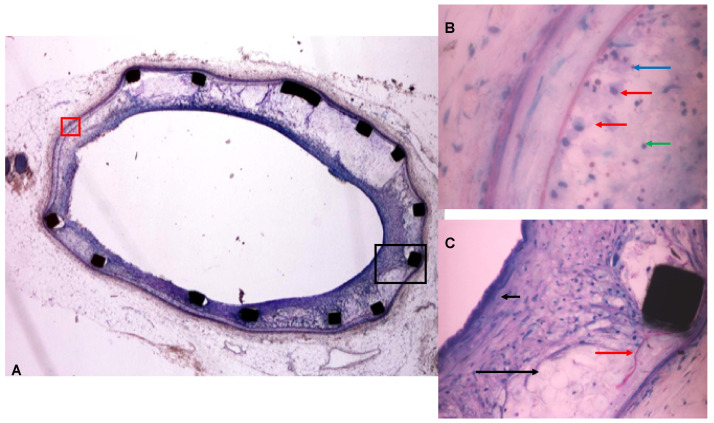
Representative picture of the control group. (**A**) This group had significantly more intimal hyperplasia and body fat deposit. (**B**) (red square; 40×): plump macrophages (red arrow), heterophils (blue arrow), Lymphocytes (green arrow). (**C**) (dark square; 20×): abundant fat bodies deposit (dark arrow), rupture of the internal elastic lamina (red arrow).

In the placebo group (Figure 1A), the stent frame was always fully incorporated into the neointima tissue whose thickness was clearly marked. In all of the specimens, the neointima was infiltrated by cholesterol clefts in a range of slight to marked grade (mean score of 2.5; Figure 1B), by macrophages and foam cells in a range of moderate to marked number with few lymphocytes (Figure 1C). The fat body deposit and the cellularity observed in group B were increased as compared to group A. These cellular and fatty components were overlaid by an endothelialized fibrous cap. New capillaries were observed in the neointima tissue. Specimens from the control group displayed an average of moderate thinning of the media in which five out of eight arteries showed some punctual lamina rupture (Figure 1C). The media were occasionally infiltrated with fatty bodies. No specific abnormality occurred in the adventitia.

**Figure 2:**
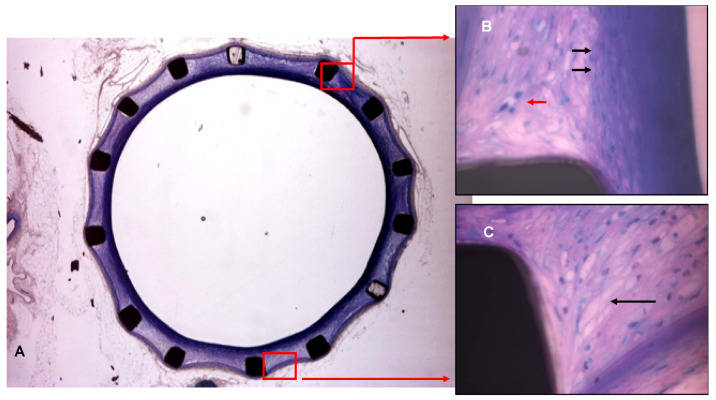
Representative picture of the treated group (Provinols™) showed a reduced neointima thickness (**A**) with clearly less inflammatory cells (**B**) and less atherosclerotic plaque deposit (**C**) when compared with the untreated group. (**B**) (40×): macrophages (red arrow), myofibroblast-like cells (dark arrow).

In Provinols™-treated animals (Figure 2A), the stent frame was well integrated into a neointimal tissue the thickness of which was obviously moderate. The neointima was infiltrated by cholesterol cleft deposits in a range of slight to moderate magnitude (mean score of 1.5), by macrophages and foam cells in range of slight to moderate number. Endothelialized fibrous caps of moderate layer thickness showing a limited collagen content overlie these cells and fat body elements. Few smooth muscle cells were occasionally encountered within the neointimal tissue. Specimens from the Provinols™-treated group displayed an average of moderate thinning of the media with no visible infiltration. Finally, no specific abnormality occurred in the adventitia.

The histopathologic readings of both groups showed in almost all specimens the presence of an atherosclerotic plaque with a fibrous cap, leading however to a lower luminal narrowing in Provinols™-treated rabbits (i.e., moderate grade) compared to the control group (marked grade).

### 3.3. Histomorphometric Quantitative Analysis

**Figure 3:**
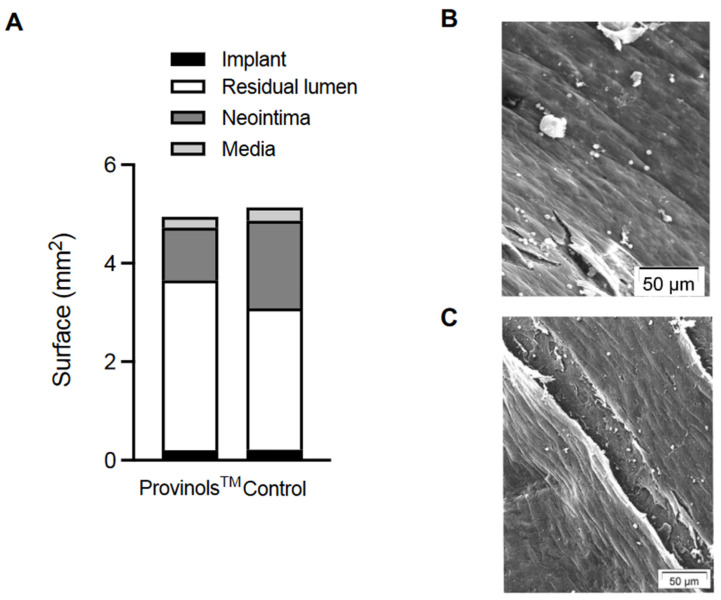
Cumulative histogram of different measured parameters (**A**). Electron microscopy of confluent endothelial cells overlying the neointimal tissue in the control (**B**) and Provinols™-treated group (**C**).

As compared to arteries from control groups, Provinols™ treatment was associated with a significant reduction of neointima thickness (44%, *p* < 0.0014), neointima surface (40%, *p* < 0.0005), with a (20%, *p* < 0.05) increase in residual lumen surface. These effects resulted in a (33%, *p* < 0.0022) significant reduction of in-stent restenosis (Table 2, Figure 3A). There was no difference in the area within the internal elastic lamina between the groups and consequently Provinols™ was not associated with either positive or negative arterial remodeling. Thus, the mean medial surface was 0.22 ± 0.1 mm^2^ in Provinols™ versus 0.27 ± 0.13 mm^2^ in placebo-treated animals. Control and Provinols™-treated arteries showed normal confluent endothelial cells overlying the neointimal tissue (Figure 3B,C).

Semi-quantitative analysis showed a marked reduction in fat body deposit (40%, *p* < 0.001) and in inflammatory cells (40%, *p* < 0.001) in Provinols™-treated animals versus placebo with a slight but non-significant (NS) hypocellularity (20% of decrease).

## 4. Discussion

The present study provides evidence that oral administration of Provinols™, a polyphenolic extract from red wine, reduced the development of intimal thickening and thus restenosis in a rabbit cholesterol-fed model of arterial injury after stent implantation. Histomorphometric analysis showed that the effect of Provinols™ was associated with the reduction of both fat deposits and inflammatory cells within the artery despite the presence of atherosclerotic plaque with fibrous cap.

Most pharmacological studies, with systemic administration of drugs, have previously failed to prevent in-stent neointima growth, failure generally attributed to an insufficient antiproliferative effect or to inadequate drug concentration at the site of injury. These unsatisfactory results led to the development of treatment at the site of the lesion using local drug delivery such as brachytherapy and DES. Intracoronary brachytherapy resulted in significant reduction of restenosis at 6 to 12 months; however, an increased rate of late recurrences in the irradiated arteries still occurs in the long term [24]. Early data with DES were very promising in prevention of restenosis. However, long term efficacy of the system greatly differs with regard to pharmacological agents, polymers coating and stents themselves (material used, design, etc.). Moreover, animal studies have shown that delayed restenosis and/or thrombosis could impair the early benefit of DES. As a result, clinicians are still left with a mandatory association of a powerful antiplatelet regimen in the long run and the risk of bleeding. Lastly, the treatment with DES remains local with high-cost incidence in patients with multi-vessels disease or even in a one-vessel disease patient in case of multiple significant lesions. For all these reasons, adjunctive or substitutive oral treatment remains a method of restenosis prevention. Hausleiter and co-workers reported beneficial effects of an oral adjunctive sirolimus treatment with intensified loading regimen in patients with angiographic in-stent restenosis [16]. Oral everolimus (a macrolide of the same family as sirolimus) has been shown to suppress in-stent neointimal growth in rabbit iliac artery [25]. Recently, dual therapy of antiplatelet drugs and the FXa inhibitor rivaroxaban in DES implantation reduced neointimal thickness and shortened the time course of neointimal growth in pigs [12]. These data were obtained in coronary-healthy animals. Probucol inhibits intimal thickening in balloon-damaged arteries of rabbit; however, in-stent restenosis was not assessed [15]. The present study was designed to investigate whether Provinols™ was able to inhibit angioplasty-induced neointimal growth after stent placement, and this in an experimental model of hypercholesterolemia.

Although the assessments of optimal drug dosage, treatment duration and pharmacological formulation need further investigation, we provide evidence that the dose of Provinols™ used (20 mg/kg) produced a sufficient amount of circulating polyphenols to reduce neointimal hyperplasia after stent placement, similar to that seen with oral everolimus [24] or sirolimus-eluting stents in animal studies [25].

Provinols™, apart from its clear efficacy, displayed a safety profile as shown by comparable weight gain, blood pressure, and biological measurements. We have reported a wide range of protective effects of polyphenols in relation to metabolic and cardiovascular diseases from in vitro and in vivo studies [26]. The molecular identity of polyphenols responsible for the in vivo effect was still unknown, but they may include oligomeric condensed tannins and anthocyanins and importantly delphinidin [27]. In our previous study, Provinols™ used in the same range of doses accelerated blood pressure lowering [28] or prevent the development of hypertension in NO-deficient and aldosterone-salt rats [29,30]. These effects of Provinols™ were associated with improved structural and functional cardiovascular changes. In the present study, the protocol was intended to analyze primarily the anti-proliferative effect of Provinols™ even though it also allowed observation of its inhibitory effect on fat body deposit, such as reduced lipid deposition. Indeed, Provinols™ has been administrated from the day of stent implantation until sacrifice at a time when the hypercholesterolemia status of the rabbits was already well established. The mechanism involved in this antiproliferative effect of Provinols™ has not been assessed. Nevertheless, our previous in vivo study in rats showed that this compound is a potent activator of endothelial NO-synthase activity and can enhance its expression in both the heart and the aorta [28,29]. Although the endothelial cells overlying the neointimal tissue were normally confluent in both groups, it is likely that the endothelium of the Provinols™ groups, but not the controls, is functional and releases NO in line with our previous results [19,20,31]. Consequently, it improves both endothelial dysfunction and vascular hyperreactivity, decreasing arterial wall thickening and fibrosis in an experimental model with vascular smooth muscle cell proliferation [28,29]. This mechanism might be advocated as NO is able to antagonize major processes involved in atherogenesis and restenosis such as platelet adherence and aggregation, monocyte activation and chemotaxis, and finally vascular smooth muscle cell proliferation. We have identified the molecular mechanisms involved in the regulation of vascular reactivity by RWP and anthocyanins. Provinols™ and the anthocyanin delphinidin modulate vascular relaxation mainly through the estrogen receptor α (ERα) [31]. Polyphenols including Provinols™ interact with ERα to activate the sirtuin-1, AMP-activated protein kinase network. Stimulation of SIRT1 and AMPK results in the activation of PPARγ coactivator 1 α, placing mitochondria at the epicenter of targets for polyphenols in CVD and metabolic disorders. In the present study, it might be possible that ERα might be a key target for the ability of Provinols™ to reduce neointimal growth in cholesterol-fed rabbit. Thus, Provinols™ might possess additional properties apart from NO to explain its beneficial effects in neointimal hyperplasia after stent placement. Reduced antioxidant and anti-inflammatory status [32] in addition to the increased NO production might work in a synergistic manner and contribute to anti-inflammatory properties of Provinols™ in vivo. These properties may be involved in the regulation of inflammatory cytokines, adhesion molecules, and chemokine production by inhibition of NFκ-B [33] or TGF-ß [34]. These later properties of polyphenols might explain the reduced inflammatory cells (macrophages, foam cells and lymphocytes) in the neointima of the iliac artery from Provinols™-treated rabbits.

Turning to vascular smooth muscle cells, their proliferation and migration are critical events for the progression of intimal thickening and the development of arterial in-stent restenosis. Several studies have reported that red wine polyphenol inhibited both processes of proliferation and migration, and this independently of NO production (for a review, see [26]). The anti-proliferative properties interfere with the cell cycle through two distinct mechanisms, down-regulation of either cyclin A gene expression or phosphatidylinositol 3′-kinase [35]. In addition, inhibition of PDGF ß receptor [36] or reduction of overexpression of vascular endothelial growth factor induced by PDGF and other growth factors [37] may account for the anti-proliferative effect of Provinols™.

## 5. Conclusions

Our study provides one of the first successful examples of prevention of neointimal hyperplasia after stent placement with a simple orally given treatment of well-tolerated Provinols™ in rabbit fed with a high-cholesterol diet. This may open a new avenue for further animal experimentations and clinical studies. The reduction of neointimal hyperplasia is indeed interesting by itself, even though further studies are needed to better understanding the underlying mechanisms. Nevertheless, the potential of the compound studied here deserves attention because if clinical studies confirm these observations, the anticipated reduction in costs will be dramatic. This study suggests a basis for the beneficial effects of red wine polyphenols against in-stent restenosis and provides robust arguments to explain the benefits linked to the Mediterranean diet.

## Figures and Tables

**Table 1:** Biochemical and physiological measurements.

	Baseline	Before Stenting		Before Euthanasia	
		Provinols™	Control	Provinols™	Control
Creatinine (µmol/L)	90 ± 20	107 ± 52	107 ± 64	122 ± 42	111 ± 26
AP (UI)	115 ± 35	131 ± 47	140 ± 46	113 ± 27	115 ± 50
GGT (UI)	12 ± 15	17 v 16	13.5 v 12	42 ± 36	80 ± 51
ASAT (UI)	15 ± 10	29 ± 9	19 ± 6.5	23 ± 3	26 ± 9
ALAT (UI)	33 ± 15	17 ± 16	13.5 ± 12	27 ± 6	41 ± 16
Bilirubin (µmol/L)	3 ± 1	4 ± 1.7	3 ± 0.6	5.1 ± 3	4.1 ± 1.2
Chol (mmol/L)	2.2 ± 1.2	50 ± 14	47 ± 10	32 ± 9	31 ± 5
TG (mmol/L)	0.87 ± 0.4	2.3 ± 2	1.35 ± 0.7	1.3 ± 0.3	2.1 ± 0.6
HDL-c (mmol/L)	0.55 ± 0.15	11 ± 1.6	10.6 ± 2.4	8.6 ± 3	8.5 ± 3.5
Glycemia (mmol/L)	11.1 ± 2.1	8.9 ± 1.4	10.4 ± 2.1	8.5 ± 1.9	9.7 ± 2.2
BP (mmHg)	----	111 ± 8	116 ± 6	110 ± 16	114 ± 10
Body weight (Kg)	3.31 ± 0.12	3.06 ± 0.45	3.03 ± 0.21	3.49 ± 0.15	3.27 ± 0.12

GGT: gamma-glutamyl transpeptidases; ASAT: aspartam amino transferase; ALAT: serum alanine amino transferase; AP: alkaline phosphatases; Chol: cholesterol; TG: triglycerides; HDL-c: high-density lipoproteins; BP: blood pressure.

**Table 2:** Histomorphometric analysis in rabbits treated with Provinols™ versus control.

	Provinols™	Control	*p*
Stenosis (%)	28.1	42.2	0.0022
Neointima thickness (μm)	179 ± 62	315 ± 153	0.0014
Neointima surface (mm^2^)	1.07 ± 0.37	1.8 ± 0.67	0.0004
Residual lumen surface (mm^2^)	3.45 ± 1	2.87 ± 1	0.05
Medial area (mm^2^)	0.22 ± 0.10	0.27 ± 0.13	NS

## Data Availability

All data that support the findings of this study are available from the corresponding authors upon request.
